# The construction of “Taishang zuo-zhe zhuxituan” in mandarin: a Cardiff grammar approach

**DOI:** 10.3389/fpsyg.2025.1553242

**Published:** 2025-04-11

**Authors:** Dajun Xiang

**Affiliations:** School of Foreign Studies, Jishou University, Jishou, China

**Keywords:** Taishang zuo-zhe zhuxituan, Mandarin, Cardiff grammar, participant role, functional syntax, functional motivation

## Abstract

Based on Cardiff Grammar, this paper studies the construction of “Taishang zuo-zhe zhuxituan” (“There is the presidium sitting on the stage”) in Mandarin, aiming to answer three key questions: (i) What Process type and Participant Roles are expressed by the construction? (ii) What is the functional syntactic structure of the construction? (iii) What are the functional motivations for using the construction? Guided by the principles of functional linguistic description, this study reveals that this construction realizes a Locational Relational Process, with two Participant Roles of Location and Carrier, which is different in meaning from similar constructions like “Zhuxituan zuo zaitaishang” (“The presidium sits on the stage”) and “Zhuxituan zaitaishang zuo-zhe” (“On the stage, the presidium sits”). The functional syntax of the construction is “S ^ P ^ C,” with the Subject conflated with Location, Complement with Carrier, and *zhe* following the verb *zuo* is an Aspect particle, which is part of the Predicator. The functional motivations of the construction include making *taishang* the Subject Theme, setting the scene for the discourse, and enhancing the state of existence of *zhuxituan*. This paper has implications for the study of other “location + V-zhe + thing” constructions in Mandarin and some similar linguistic phenomenon in other world languages.

## 1 Introduction

The construction of “Taishang zuo-zhe zhuxituan” (台上坐着主席团, “There is the presidium sitting on the stage”) in Mandarin has garnered substantial scholarly attention in the field of Chinese linguistics since the 1950s (Lü et al., 1956). To elucidate the phenomenon, we shall examine this construction closely, as illustrated in (1)^1^.

**Table 1:** 

(1)	台上	坐	着	主席团。
	Taishang	zuo	zhe	zhuxituan.
	stage-on	sit	ASP	presidium.
	“There is the presidium sitting on the stage”			

This construction expresses the meaning of “there exists a thing at some places” with the pattern of “location + V-zhe + thing,” including three components: a locative *taishang* (“on the stage”), a verb *zuo* (“sit”) followed by an aspect particle *zhe*^2^, and a noun *zhuxituan* (“the presidium”) in that order. The construction has been deemed “noteworthy” in the Chinese linguistic literature due to the semantic and syntactic mismatch it has exhibited. It is generally argued in the literature that the construction logically means *zhuxituan zuo-zhe* (“The presidium sits”) (Peng, 2018: 200; Lü, 2002: 302; Wang, 2002: 374). In other words, the logical Subject of the verb *zuo*, i.e., *zhuxituan*, which should conventionally occupy the initial position of the clause, is instead found at the end, while *taishang*, typically an adjunct in Mandarin, assumes the Subject position. However, this construction seems not to be a solitary case but a common one in Mandarin because a number of other verbs can also enter into this construction, such as *zhan* (“stand”), *tang* (“lie”), *zhu* (“live”), *gua* (“hang”), etc. There are 5,372 tokens with the pattern of “location + V-zhe + thing” in the BCC (Chinese Corpus constructed by Beijing Language and Culture University Corpus Center). Here are some similar expressions:

**Table 2:** 

(2)	墙上	挂	着	一幅画。(BCC)
	Qiangshang	gua	zhe	yifu hua.
	wall-on	hang	ASP	a picture.
	“There is a picture hanging on the wall.”

**Table 3:** 

(3)	窗外	飘	着	雪。(BCC)
	Chuangwai	piao	zhe	xue.
	window-outside	drift	ASP	snow.
	“There is the snow drifting outside the window.”			

Owing to these distinctive characteristics, this construction is frequently cited as a quintessential example of “locative inversion” constructions, presenting significant challenges for grammatical description and elucidation within the field of Chinese linguistics. The scholarly discourse has seen a variety of theoretical frameworks put forth for the syntactic and semantic analysis of this construction, with a particular focus on the grammatical function of the locative *taishang*, viz. whether it can be analyzed as the Subject of the clause (Lü et al., 1956; Song, 1991; Li, 2001; Zhu, 2001; Lü, 2002; Wang, 2002; Wang and Xu, 2013; Li, 2011; Deng, 2016; He, 2016; Wang, 2016; Yang, 2019; Xu and Pan, 2019). Although new proposals are emerging, the problems remain unresolved. However, little attention has been paid to the particular insights into such a construction that can be obtained from an analysis by drawing on Cardiff Grammar (CG), a model of Systemic Functional Linguistics (SFL). The purpose of this paper is to put forward this kind of analysis.

In this paper, the construction of “Taishang zuo-zhe zhuxituan” will be studied within the framework of CG (Fawcett, 1980, 2008, 2010, 2011, 2013, 2017; Tucker, 1998; He et al., 2015)—a cognitive-interactive model of SFL. From the perspective of CG, the crux of the matter in this construction is insufficient linguistic description and explication in Mandarin. Through functional semantic and syntactic analysis, this paper aims to answer three specific questions related to this construction: (i) What Process type and Participant Roles are expressed by the construction? (ii) What is the functional syntactic structure of the construction? (iii) What are the functional motivations for using the construction? By addressing these three pivotal questions, we aim to show that the framework of CG can illuminate the intrinsic nature of this construction both theoretically and practically.

The subsequent sections of this paper are organized as follows. Section 2 makes a sketch of the previous studies of this construction, where different viewpoints will be reviewed critically. Section 3 provides a brief overview of CG with the purpose to prepare the ground for the analysis of the construction. Section 4 focuses on the process and Participant Role (PR) analysis of this construction. Section 5 delves into the functional syntax of the construction. Section 6 investigates the functional motivations underlying the use of the construction, and Section 7 synthesizes the findings and presents the concluding remarks.

## 2 Previous studies of “Taishang zuo-zhe zhuxituan”

The construction of “Taishang zuo-zhe zhuxituan” has often been treated as one particular kind of existential constructions in the Chinese linguistic literature. Since the period of “Subject and object discussion” in the 1950s (Lü et al., 1956), it has interested many scholars from different branches of linguistics, including traditional grammar, formal linguistics, cognitive-functional linguistics and linguistic typology to determine which element serves as the Subject in the construction. Since convergence and divergence are identified among different linguistic branches, we will review previous studies from various perspectives on the analysis of *taishang* in the construction. Generally, there are four views: the adjunct view, the Subject view, the Big Subject view, and the complement view. Each view encompasses distinct rationales. Given the comprehensive nature of previous studies, this section will elucidate each view with resort to representative figures.

### 2.1 The adjunct view

The Adjunct view holds that *taishang* is an Adjunct. It is argued that locatives in Mandarin are adjuncts, as they typically lack semantic relations whatsoever with the verbs of the clauses. Two sub-types are found within this view: View A and View B. The former claims that the construction has the syntactic structure of “A ^ P ^ S,” i.e., *taishang* is the Adjunct, *zuo-zhe* is the Predicator^3^, and *zhuxituan* is the Subject (Peng, 2018: 200; Lü, 2002: 302; Wang, 2002: 374; Chen et al., 2023). The latter argues that its syntactic structure is “A ^ P ^ C,” that is, *taishang* is the adjunct, *zuo-zhe* is the predicator, and *zhuxituan* is the complement^4^. There is no Subject in this construction (Sun, 1958; Gao, 1986; Wang, 2016).

View A is fundamentally grounded in the semantics among the three elements of the construction, with a focus on the “Agent-Affected (‘patient’ in other grammar)” relationship. The Predicator *zuo-zhe* is semantically linked exclusively to the animate Agent *zhuxituan* rather than *taishang*, indicating that the action denoted by *zuo-zhe* is attributed to *zhuxituan*. Thus, it is the Subject. However, this Subject is not a conventional one. It is a “dependent Subject” because it is contingent upon the Predicator *zuo-zhe* (Wang, 2002: 374). Consequently, View A is occasionally referred to as the “semantic school.” It posits that the construction is semantically equivalent to *Zhuxituan zuo zaitaishang* (“the presidium sits on the stage”), with *taishang* being thematized, i.e., an inversion of *Zhuxituan zuo zaitaishang*. This view is to some extent functionally oriented. Nevertheless, the problems with this view are also apparent. One is that it ignores the semantic role of *taishang*, not accounting for its obligatory presence, which contradicts “S ^ P ^ C” tendencies in Mandarin. The other is that the traditional concept of inversion must be redefined, given that the standard definition of inversion is the movement of an element from its canonical position to a non-canonical one without altering the constituent elements (Quirk et al., 1985: 1377–1379). In other words, scholars must prove that *zuo-zhe* and *zuo zai* (“sit at”) are semantically equivalent. In fact, the placement of the Agent at the end of the construction is a prevalent rule in Mandarin (Lü, 2002: 523; Li, 2011: 111).

View B posits that the construction in question is an existential clause, a notion distinct from the English language, which necessitates a semantically empty Subject *there*. Unlike English, Mandarin does not mandate the presence of a Subject in existential clauses. This view categorizes the construction as a non-Subject clause, reflective of Chinese characteristics. However, this view encounters two challenges. One is that it lacks compelling evidence to categorize a substantial number of such clauses in Mandarin as non-Subject clauses. The other is that the construction diverges significantly in both form and meaning from prototypical non-Subject clauses, exemplified by *Xia Yu le* (“It rains”), where there is no Subject, and *yu* (“rain”) is a Complement rather than a Subject. Consequently, the functional validity of View B is also open to scrutiny, which lacks empirical support.

To summarize, the Adjunct view is profoundly rooted in the traditional grammar, where a rigid one-to-one correspondence between clause elements and word groups has been unconsciously maintained, including a direct mapping from subjects to nominal groups, Adjuncts to prepositional groups (phrases), complements to adjective groups, and so forth (Quirk et al., 1985: 60). Given that locatives in Mandarin bear a close semantic resemblance to prepositional groups, they are consequently categorized as Adjuncts.

### 2.2 The subject view

The Subject view asserts that *taishang* functions as the Subject within a syntactic structure of “S ^ P ^ C” (Song, 1991: 119–120; Zhu, 2001: 408–409; Yang, 2019). This view, often referred to as the “word order school” or “positionalism,” primarily relies on linguistic form, specifically word order, to identify Subjects. According to this criterion, if a nominal group precedes the verb, it is a Subject; conversely, if it follows the verb, it is deemed the complement. Zhu (2001: 411) claims that the challenge of identifying a clause element as a subject or complement hinges on discerning whether the structure containing the element is “subject-predicate” or “predicate-complement.” It is claimed that subjects and complements in Mandarin are less influenced by semantic roles and are more determined by their structural positions. This view further argues that temporal and locative elements share formal features with Agents, suggesting they should be categorized as the same grammatical category. Given that the locative *taishang* precedes the verb *zuo* in the construction, it is considered a special noun, and by extension, a Subject.

The principal advantage of the Subject view lies in its simplicity and ease of application in identifying Subjects and Complements, aligning with the traditional definition of the Topic as the Subject (Chao, 1968: 69; Song, 2018: 200). However, the shortcomings of this view are equally evident. Wang (2002: 367), from the “semantic school,” critiques this view for its exclusive focus on linguistic form, which obscures the boundary between rhetoric and grammar. The consequence is that the diverse and vibrant expressions in Mandarin have been restricted into a set of rigid rules. For instance, treating the underlined item *mingtian* in *Mingtian yao kaihui* (“Tomorrow there is a meeting”) as a Subject contradicts the conventional grammatical description in Mandarin, where it is typically regarded as an Adjunct.

In essence, the Subject view overemphasizes word order, treating *taishang* as Subject solely due to its position, while neglecting its semantic role as Location. This leads to inconsistencies as stated above.

### 2.3 The big subject view

The Big Subject view posits that the construction is a “subject-predicate predicate clause,” where *taishang* is identified as the Big Subject, and *zhuxituan* serves as the Little Subject within the clause *zuo-zhe zhuxituan* (literally “sit the presidium”) (Li, 2001: 342–345). This view suggests that the “subject-predicate” structure of *zuo-zhe zhuxituan* functions as the predicate of the Big Subject *taishang*. Thus, the proposed syntactic structure of the construction is “S_big_ ^ P ^ S_little_,” with an inversion of the Little Subject and the verb.

In this view, *taishang* is deemed the Subject of the main clause, and *zuo-zhe zhuxituan*, regardless of its internal structure, i.e., *zuo-zhe zhuxituan* or *zhuxituan zuo-zhe* (“The presidium sits”), operates as its predicate. Functionally, this view delineates the psychological subject (Theme in SFL) as the big subject and the logical subject as the little subject. The core tenet of this view is that topics in Mandarin equate to subjects. This view shares an advantage with the subject view in that it aligns with the traditional definition of the topic as the subject. However, two issues require resolution. On the one hand, this view does not consider the semantic interrelations among the three elements of the construction within the broader syntactic pattern, recognizing only the semantic connection between the verb *zuo* and the noun *zhuxituan*, while denying the semantic relations between the Predicator *zuo-zhe* and the locative *taishang*. This stance partially aligns with View A in the Adjunct view, which also regards *zuo-zhe zhuxituan* as an inversion of *zhuxituan zuo-zhe*. On the other hand, this construction diverges from the typical “subject-predicate predicate clauses” (see He, 2017), exemplified by *Ta shenti jiankang* (“He is physically healthy”), where the embedded clause *Shenti jiankang* (“The body is healthy”) adheres to “subject + predicate” structure. This divergence implies a significant expansion of the “subject-predicate predicate clauses” in Mandarin.

In a word, functionally a topic is a semantic (pragmatic) concept (Lü, 2002: 616) and does not occupy the same categorical level as the subject and the complement. Although related, they are distinct entities. Thus, equating the topic as the subject may lead to confusion between semantic and syntactic structures.

### 2.4 The complement view

The Complement view claims that *taishang* in this construction functions as a complement, with *zhuxituan* assuming the function of the subject within a syntactic structure characterized as “C ^ P ^ S” (Deng, 2016). This view shares certain affinities with the Adjunct view, where *taishang* is deemed ineligible for Subject status, and *zhuxituan* is recognized as the indisputable subject. However, diverging from the adjunct view, the complement view regards *taishang* as an inherent syntactic element, i.e., a complement, instead of an adjunct. This stance admits the semantic relationships between *taishang* and the verb *zuo*, yet demurs from recognizing it as the subject, given that *zhuxituan* fulfills the role of agent. Functionally, this view echoes the traditional grammar, where subjects are often agents, and agents are accorded precedence in subject assignment.

The advantage of this view is its recognition of *taishang* as an inherent syntactic element, realizing a PR. Nonetheless, it overlooks the basic syntactic sequence “S ^ P ^ C” in Mandarin, where agents constitute only half of the subjects within clauses (Shen, 2017). Other elements such as time, locatives, tools, and so forth, are also capable of serving as subjects. Thus, while this view has a functional orientation, its functional scope is unduly constricted, effectively confining the subject to agents alone, which may ignore Mandarin’s flexibility in assigning subjects to locatives and temporals.

All in all, the above views diverge in their interpretations of the nature of the construction, particularly concerning the locative *taishang*, and none of these competing views has gained the most ground for the time being. To a certain extent, the adjunct view and complement view are meaning-oriented, whereas the subject view and the big subject view are form-oriented. Although all these views claim that they have taken both form and meaning into consideration, i.e., the long-recognized methodological principle of “integration of both form and meaning” within Chinese linguistic tradition (Zhu, 2001: 146; Fan, 2018), the ongoing debate surrounding the construction of “Taishang zuo-zhe zhuxituan” highlights the lack of consensus on implementing this principle, especially the unresolved issue of mutual verification between form and meaning. All these indicate that a new approach focusing on the perspective of semantic-and-syntactic alignment is needed to shed light on the very nature of the construction in question. Distinct from other grammatical approaches, CG regards the system network as the generative base of a linguistic structure. Therefore, meaning and function, and their syntactic realization have become the main focus of this grammar. A CG approach is capable of not only uncovering the semantic and functional features of the construction, but also elucidating the distinctions between this construction and analogous ones. Prior to delving into this construction, we will provide a concise overview of the basic notions within CG to establish the theoretical framework.

## 3 A brief overview of Cardiff grammar

CG has its basis in SFL theory and, in particular, in Halliday’s earlier work in the 1960s and 1970s (Schulz and Fontaine, 2019: 230). As a cognitive-interactive model within SFL (Fawcett, 2008: 20), CG places greater emphasis on the role of the “interacting mind” in constructing a model of language and its use, striving to achieve a balance between the descriptive, generative, and cognitive aspects of language. This section aims to delineate the basic notions of CG for the purpose to provide an analytical framework for this study. Three pivotal aspects will be specified: the relationship between meaning and form, the basic principles that underpin the analysis of Processes and PRs, and the basic syntactic categories and relations.

### 3.1 The relationship between meaning and form in CG

Within the framework of cognitive-functional linguistic theories, CG shares a foundational commitment to explicating linguistic structures through bidirectional form-meaning correlations. Following Saussurean duality principles, Fawcett’s (2008: 37) and Fawcett’s (2010: 34) theoretical premise maintains that linguistic signs inherently possess dual aspects: formal manifestations and semantic content, proposing an inseparable dialectic where formal analysis necessitates concurrent semantic consideration. This conceptualization extends beyond conventional semantic parameters in SFL, particularly through Halliday’s functional expansion where three meta-functions proliferate into eight distinct meaning dimensions within CG: experiential, interpersonal, thematic, logical relationships, polarity, validity, affective and informational (Fawcett, 2008: 242).

**Figure 1:**
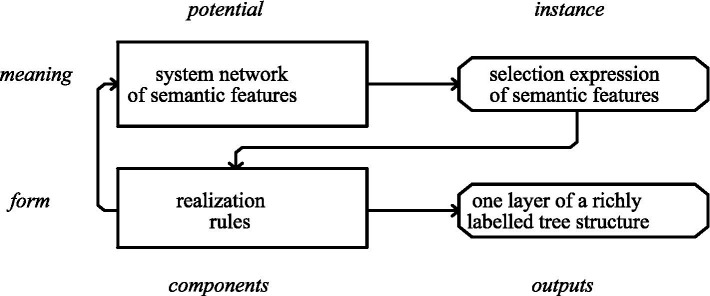
The components and their outputs in a systemic functional grammar (Fawcett, 2008: 41).

CG operationalizes paradigmatic analysis through semantic opposition rather than formal differentiation, positing that forms are meaning realizations. The model’s organizational axiom establishes the formal stratum as encompassing all meaning-realization mechanisms, including syntax, items, intonation, and punctuation. Furthermore, CG theoretically distinguishes between language as a systemic resource (the potential) and its contextualized implementation (instantiated text), with instantiation processes mediating between these ontological levels. This form-meaning dynamic finds visual representation in Figure 1, illustrating their interdependent relationship within the CG paradigm.

In Figure 1, the boxes under the label “potential” are concerned with the language as a system, while those labeled as “instance” refer to the products of particular uses of that system, i.e., texts, defined as instances of language in use. In this model, lexicogrammar has two main components that specify “potentials” (on the left), and two outputs that specify “instances” (on the right). Therefore, the system network of semantic features specifies the language’s meaning potential, and the realization rules specify its form potential, their output being a syntactic unit and its elements. As it shows, the result of instantiation at the level of meaning is a semantic selection expression, consisting of all the features selected from the semantic networks in the generation of a particular clause, and the results of instantiations at the level of form are the complete structures for the units under generation. The two levels of form and meaning are connected through the fact that the output from the level of meaning is the input to the level of form. If an element of the generated unit needs to be filled by a further unit, a realization rule (represented by the arrow on the left) specifies re-entry to the network to generate one.

### 3.2 Basic principles in the analysis of processes and PRs

Within the meaning potential, a pivotal domain is the network for TRANSITIVITY, which encompasses two key concepts: (i) Process, which is typically realized in the Main Verb of a clause, and (ii) Participant Roles (PRs), which are roles that are “expected” by the Process, and are typically conflated with the Subject or the Complement. CG has proposed several principles when analyzing Processes and PRs (see Fawcett, 2011):

(i) There must be no more than one of each type of PR in any one clause;
(ii) There must be a test for each PR, to enable the analyst to check in cases of doubt;
(iii) The same configuration of PRs is not used for more than one major Process;
(iv) In identifying the Processes or PRs, PRs should come first.

CG delineates six principal processes within the TRANSITIVITY system: action process, relational process, mental process, environmental process, influential process, and event-relating process. Each of these processes is characterized by distinct configurations of PRs. Within the relational process, five subcategories are identified: attributive, locational, directional, possessive and matching. Given the close relevance of the construction under discussion to the Locational Process, we will delve deeper into this particular subcategory. In CG, a Location can pertain to either space or time, which is usually “static.” Locations in space are more common, because the sort of entity that typically gets located in time is an event, and events are typically realized as clauses (Fawcett, 2010: 210). If a Location includes a preposition, that preposition is always part of the Location. According to Fawcett (2011), locational process in English is characterized by four distinct configurations of PRs as below.

- Carrier+Process+Location: Ivy[Ca] lives/is/works[Pro] in Cardiff[Loc].
- There+Carrier+Process+Location: There is[Pro] a fly[Ca] in my soup[Loc].
- Affected-Carrier+Process+Location: His luggage[Af-Ca] stayed[Pro] in Cardiff[Loc].
- Agent-Carrier+Process+Location: Ivy[Ag-Ca] remained[Pro] in Cardiff[Loc].
- Agent+Process+Affected-Carrier+Location: Ivy[Ag] kept[Pro] the baby[Af-Ca] at home[Loc].

Type A features a simple carrier and is the most frequent, accounting for 97% (Fawcett, 2011). This type includes a subcategory characterized by the “there be” construction, wherein the existential *there* does not conflate any PR. This subcategory is to present the Carrier as an “enhanced theme” in a Process that typically encapsulates a state of “being” at a certain Location in space or, less frequently, in time. Its discourse function is to introduce a new “object” to the discourse, which is why it almost invariably appears with an unparticularized (indefinite) nominal group as the carrier. Type B is distinguished from type A by having a compound PR, i.e., Affected-Carrier. In this type, the Carrier bears no responsibility for the decision that dictates its presence in the Location. Type C involves a positive decision on the part of the Carrier to maintain its state, exemplified by *Ivy* not merely being a simple carrier but also functioning as an Agent-Carrier. This compound role is capable of passing the re-expression tests for both Agent and Carrier, as pointed out by Fawcett (2011). Type D pertains to actions in the “psychological” realm, where the agent decides to keep something in a specific location. Here in this case, the location is defined by the presence of *the baby*, rendering *Ivy* the agent, and the carrier, i.e., *the baby*, not just a simple carrier but an affected-carrier.

### 3.3 Three principles for functional syntax

The most prominent feature of CG lies in its construction of a theory of syntax for SFL. Three major principles are identified for functional syntax within SFL (Huang, 2007; He, 2017; He, 2023: 359–360). The first principle is that language is multi-functional, which implies that linguistic forms reflect multi-dimensional rather than single-dimensional meanings. The second principle is that meaning is primary, and form is the realization of meaning. The cardinal principle in CG is that meaning potential is the generative basis for linguistic forms, and semantic features must be realized in linguistic forms. In other words, syntactic analysis is based on semantic analysis and is a refinement of semantic features. The third principle says that the semantic features of the semantic layer are realized by a single syntactic structure at the level of form. The implications of this principle are that elements may be conflated with each other, one element may realize more than one semantic feature, and different elements combined may realize different strands of meaning.

**Figure 2:**
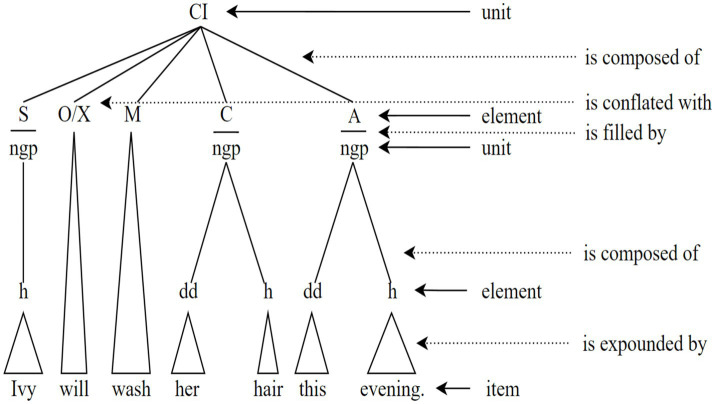
The basic categories and relations of syntax in CG (Fawcett, 2008: 75).

Based on these three principles, four functional syntactic categories and relations are posited (Fawcett, 2008, 2010). The former includes unit, element, place, and item, while the latter encompasses componence, filling, exponence, and conflation. The basic syntactic categories and relations in CG can be represented in Figure 2.

In Figure 2, this clause is composed of five elements: subject, operator/auxiliary, main verb, complement, and adjunct. The subject is filled by a nominal group, which is composed of head only and expounded by the item *Ivy*. The operator is conflated with auxiliary, expounded by the item *will*. The main verb is expounded by the item *wash*. The complement is filled by a nominal group, which has two elements, i.e., a deictic determiner expounded by *her* and a head expounded by *hair*. The adjunct is filled by a nominal group composed of two elements, i.e., a deictic determiner expounded by *this* and a head expounded by *evening*.

From the vantage point of CG, a robust functional linguistic theory must exhibit the strength at both the functional and formal levels, for the simple reason that every functional description must, if it is to be sufficiently explicit to be testable, also attend carefully to the level of form (Fawcett, 2008: 15). The result is that the purely formal contrasts in a language play no role in how the grammar operates in the generation of a sentence. CG provides a cognitive-interactive model of the interacting mind that also incorporates knowledge of the socio-cultural factors relevant to generating and understanding language-texts. This grammar has been and is being developed around the world and in different languages, for example, in work on Mandarin (Zhou, 1997; He et al., 2015; Yan and He, 2022), German (Schulz, 2015), and Japanese (Tatsuki, 2020). In the ensuing section, we will analyze the semantics and functional syntax of the construction of “Taishang zuo-zhe zhuxituan” from a CG approach. It will be seen that CG is appliable to Mandarin, and the seemingly irresolvable problem of this “special” construction can be properly handled with simplicity in this grammar.

## 4 The functional semantics of the construction

Within CG, the experiential strand of meaning in clauses is primarily conveyed through the system of TRANSITIVITY, which defines (i) the range of types of process that is possible to express through the language concerned and (ii) the PRs in each of those types process (Fawcett, 2008: 47). As pivotal elements for the experiential meaning, the analysis of processes and PRs provides a methodology for elucidating the perspective and worldview of the text’s performer, be it a speaker or a writer. This section first analyzes the process and PRs of the construction in question, and then makes comparisons with analogous constructions.

### 4.1 The process and PR analysis of the construction

The construction of “Taishang zuo-zhe zhuxituan” is fundamentally contingent upon the semantic interplay among its three elements: *Taishang*, *zuo-zhe*, and *zhuxituan*. If *zuo-zhe* is intransitive, it expects only one participant, whereas a transitive reading expects two participants. There is a scholarly consensus on identifying *zhuxituan* as a PR, but opinions diverge regarding *taishang*. Thus, the key to resolving this issue lies in determining the grammatical function of *taishang*—whether it is a PR or a Circumstantial Role (CR). According to CG, if it is a PR, it is expected by the Process, an intrinsic element; if it is a CR, it is not expected by the Process, an optional element (Fawcett, 2008: 137). Given that processes are typically expressed by verbs, the meaning of the verb *zuo* must be examined first. The fifth edition of the Modern Chinese Dictionary (Cao et al., 2007: 1828) indicates that *zuo* can function both transitively and intransitively, for example *zuo chuan* (“take a boat”) and *qing zuo* (“please take a seat”). This suggests that the traditional distinction between transitive and intransitive verbs in Mandarin is not always clear-cut, as verbs can exhibit both behaviors depending on contexts, as keenly noted by Shen (2018). Through corpus investigation in the BCC, it is found that there are 3,070 tokens with the pattern of “zuo-zhe + ngp” among 26,528 tokens with “zuo-zhe,” accounting for 11.6%. This suggests that this construction warrants particular consideration and should be recognized as a common pattern, as argued by Lü (2002: 523).

In CG, it is meaning rather than form that is the pivotal factor in process and PR analysis. Adhering to CG’s criteria for identifying PRs, should the Process of “zuo-zhe” expect two PRs or only one PR? In other words, should it be regarded as transitive or intransitive? This raises a very important question, i.e., in identifying the process and PRs, which should come first? Fawcett (2011) points out that “we focus first on identifying the PRs.” There are two reasons: (i) the first is that the simplest and most reliable way to identify a Process type is via its PRs; (ii) the second is that identifying the types PR is typically more revealing than identifying the type of the Process. From this point, if we regard the 3,070 tokens (11.6%) with the pattern of “zuo-zhe + ngp” as intransitive, it seems not very convincing, since *taishang* and *zhuxituan* are two “potential” PRs in the construction. Based on this observation, we claim that the Process of “zuo-zhe” in this construction expects two PRs in this particular context. Of course, we do not claim that the Process of “zuo-zhe” will always expect two PRs. In some contexts, it is indeed the case that it expects only one PR, see Section 4.2 for further discussion. Therefore, the meaning of the construction is “Moudi zuo-zhe mouren” (“somewhere sits someone”), which means that *taishang* is an intrinsic PR, not a CR, as it is expected by the Process of “zuo-zhe.” Should *taishang* be omitted from the construction, the clause (i.e., Zuo-zhe zhuxituan, “sit the presidium”) would be unacceptable, as emphasized by Song (1991: 122). Without contextual information, an Addressee would inevitably inquire “Nali zuo-zhe zhuxituan?” (“Where does the presidium sit?”), highlighting the indispensability of *taishang* in the construction. This reveals the fact that the construction of “Taishang zuo-zhe zhuxituan” is different from the construction “Zhuxituan zaitaishang zuo-zhe,” in which the Process of “zuo-zhe” expects only one PR (see Section 4.2 for further discussion).

Therefore, according to CG, the construction of “Taishang zuo-zhe zhuxituan” realizes a locational relational process, which consists of two PRs, i.e., location and carrier. The configuration of PRs in this construction is “Location + Process + Carrier,” where *taishang* expresses the location, *zuo-zhe* expresses the process, and *zhuxituan* expresses the carrier. It is important to note that in this construction the carrier *zhuxituan* is a simple carrier without an associated agent role. This is attributed to the fact that the construction is a “static” description of *taishang*, and *zhuxituan*, despite being an animate noun, does not exert any agency in maintaining this state. In essence, the construction is experientially equivalent to *Taishang shi zhuxituan* (“There is the presidium on the stage”) or *Taishang you zhuxituan* (“There exists the presidium on the stage”), as posited by Lu (2009) and Li (2011: 112). They argue that existential clauses in Mandarin can be structured by *shi* (“be”) and *you* (“have”), which also serve to describe the Location of objects (see (4)).

(4) a. Limian[Loc] shi[Pro] baozang[Ca] (“There are treasures inside”). (BCC)

b. Shuili[Loc] you[Pro] yu[Ca] (“There are fish in the water”). (BCC)

According to CG, re-expression tests are employed to ensure the reliability and validity of PR analysis. As outlined by Fawcett (2011), if X is the Location and Y is the Carrier, the clause can be re-expressed as “X is where Y was.”^5^ The construction can be re-expressed as *Taishang shi zhuxituan de suozai zhichu* (“on the stage was where the presidium was”). So, *taishang* as the PR of Location can pass the test. If X is a simple Carrier in a Locational Process, the clause can be re-expressed as “The thing about X was that…” The construction can be re-expressed as *Guanyu zhuxituan de shi shi tamen zai taishang* (“The thing about the presidium was that they were on the stage”). So, *zhuxituan* as the PR of carrier can pass the test. Some scholars contend that *zhuxituan* assumes the agent role in this construction (Peng, 2018: 200; Lü, 2002: 302; Wang, 2002: 374). In CG, the test for an agent states that if X is the Agent, the clause can be re-expressed as “What X did was to …” It is very odd for the construction to be re-expressed as *Zhuxituan suo zuo de shi zuo zaitaishang* (“What the presidium did was to sit on the stage”), since this construction does not convey this kind of meaning. This test is actually used for the construction *Zhuxiantuan zuo zaitaishang* (“The presidium sits on the stage”) (see section 4.2 below). Others argue that *zhuxituan* is a compound PR of Agent-Carrier (Deng, 2016). According to Fawcett (2011), the test for Agent-Carrier in Relational Processes can be re-expressed as “The thing about X was that … as a result.” It is quite odd to re-express the construction as *Guanyu zhuxituan de shi shi tamen zuizhong zuo zaitaishang* (“The thing about the presidium was that they sat on the stage as a result”). Therefore, the widely recognized Agent role of *zhuxituan* in this construction must be revisited. Contrary to other views, we argue that in this construction, *zhuxituan* is not a PR of Agent capable of controlling the process; instead the location assumes a more central PR. In fact, Li (2011: 113, 114) and Song (1991: 111), and many others have previously argued that the verb in this construction does not convey an action but rather a state of existence, signifying that it has relinquished “control” over the noun it is followed by, irrespective of whether it is animate or not. Among the 416 tokens with the pattern of “location + zuo-zhe + thing” in the BCC, no tokens are found with an agent “thing.” In other words, when verbs are followed by the aspect particle *zhe* in this pattern, their functions are not literal but serve to express the continuation of the state of existence.

### 4.2 Process and PR analysis of analogous constructions compared

In the literature, some linguists argue that the construction is semantically equivalent to other related clause patterns (Deng, 2015; Li, 2001: 342–345; Peng, 2018: 200; Lü, 2002: 302; Wang, 2002: 374), such as (5) and (6) below. While distinctions among these patterns have been noted, a comprehensive and targeted comparative analysis, particularly within a unified theoretical framework, has been conspicuously absent. It is the aim of this section to bridge this gap by conducting a systematic comparison that elucidates the nuanced differences among these constructions.

**Table 4:** 

(5)	主席团	坐	在台上。
	zhuxituan	zuo	zai taishang.
	presidium	sit	on the stage.
	“The presidium sits on the stage.”

**Table 5:** 

(6)	主席团	在 台上	坐 着。
	zhuxituan	zai taishang	zuo-zhe.
	at the door	at stage-on	sit ASP.
	“On the stage, the presidium sits.”		

In (5), *zaitaishang* (“on the stage”) is a prepositional group. According to CG, the Process expressed by the verb *zuo* in (5) expects two PRs. In other words, the meaning of (5) is “mouren zuo zaimoudi” (“someone sits at somewhere”), which is apt to respond to the query of *Zhuxituan zuo zai nali?* (“Where does the presidium sit?”). In this clause, *zaitaishang* is also an intrinsic PR, since the omission of it generates an unacceptable clause *Zhuxituan zuo* (“The presidium sits”), which is semantically incomplete. Therefore, in accordance with CG, example (5) expresses a Locational Relational Process, where *zhuxituan* has the PR of Agent-Carrier, and *taishang* the Location. Accordingly, the PR configuration of (5) is “Agent-Carrier + Process + Location.”

It seems that (1) and (5) share the same Process type and PRs. However, a notable distinction arises in (5), where *zhuxituan* is a compound PR of Agent-Carrier. This implies that *zhuxituan* in (5) is not merely a Carrier but also an Agent. To elucidate, we can resort to CG’s re-expression tests for an Agent and a Carrier. Example (5) can be re-expressed as *Zhuxituan suo zuo de shi zuo zaitaishang* (“What the presidium did was to sit on the stage”) and *Guanyu zhuxituan de shi shi tamen zaitaishang* (“The thing about the presidium was that they were on the stage”), thereby confirming *zhuxituan* as both an Agent and a Carrier. In accordance with Fawcett’s (2011) further test for agent-carrier in relational processes, the clause can be re-expressed as *Guanyu zhuxituan de shi shi tamen zuizhong zuo zaitaishang* (“The thing about the presidium was that they sat on the stage as a result”). Thus, *zhuxituan* in (5) is indeed not just a simple carrier, but an agent-carrier. This suggests that *zhuxituan* in (5) as an animate noun involves a positive decision on the part of the Carrier to maintain the state, such as choosing to sit rather than stand, or on the stage instead of elsewhere. From the perspective of CG, the distinction between (1) and (5) lies in the meanings of the processes realized by the predicators *zuo-zhe* and *zuo, respectively.* Despite the fact that they express the same process of location, they expect different PR configurations. At this juncture, it is plausible to assert that *zuo-zhe* is distinct from *zuo*. When the verb *zuo* and its subsequent element become semantically unbalanced, they necessitate contextual adjustment and re-organization. This underscores the notion that word order is an intrinsic aspect of linguistic form in Mandarin.

According to CG, *zuo-zhe* in (6) expects a solitary PR, thereby conveying the meaning “Mouren zuo-zhe” (“someone sits”), which is likely to respond the query *Zhuxituan zainali zuo-zhe?* (“Where does the presidium sit?”). According to CG’s process types (Fawcett, 2011), it realizes an action process, with *Zhuxituan* realizing the mere PR of agent. Consequently, the PR configuration for (6) is succinctly characterized as “Agent + Process.” To validate the role of *zhuxituan* as an agent, CG’s re-expression test can be applied. The clause can be re-expressed as *Zhuxituan suo zuo de shi zuo-zhe* (“What the presidium did was to sit”). So, as an Agent, *zhuxituan* can pass the test successfully in (6). It is important to note that in this clause, *zaitaishang* is a CR, not a PR, as it is not expected by the Process of “zuo-zhe.” The omission of this CR does not render the clause meaningless, thereby preserving the coherence of *zhuxituan zuo-zhe* (“The presidium sits”). There may be a variant form of (6), such as *zaitaishang zhuxituan zuo-zhe* (“on the stage, the presidium sits”). Here, *zaitaishang* is still a CR, which enjoys a degree of syntactic flexibility in the clause, akin to its counterpart in English. Therefore, the analysis suggests that (6) is also distinct from (1), not only in process type but also in PRs.

To sum up, the process and PR analyses of (5) and (6) reveal that they are different from (1), indicative of unique constructions. This proves the fact that distinct structural patterns map divergent semantic contents (Hunston and Francis, 2000). Therefore, the problem for scholars who equate the above clauses is that they ignore the different shades of meaning constructed by disparate patterns. From the perspective of CG, these varied syntactic patterns, replete with distinct Processes and PR configurations, serve as potent tools for generating a spectrum of meanings and performing a variety of functions in a range of linguistic contexts.

## 5 The functional syntax of the construction

Based on the three major principles proposed by CG for functional syntax as stated in Section 3, this section is devoted to the functional syntax of the construction, aiming at providing a systematic syntactic description consistent with the meaning it expresses. Before our functional syntactic analysis of the construction, it is imperative to establish a lucid comprehension of the concept of Subject in Mandarin.

### 5.1 Subject and its identification

The term Subject is a basic concept to the Western tradition of grammatical analysis. Various interpretations have developed around the Subject notion, ascribing to it a number of rather different functions, which can be summarized below:

(i) that which is the concern of the message;
(ii) that of which something is being predicated;
(iii) the doer of the action.

These three definitions are obviously not synonymous; they are defining different concepts. In the second half of the nineteenth century, when there was a renewal of interest in grammatical theory, three terms came to be used, that is, psychological Subject, grammatical Subject, and logical Subject (Halliday and Matthiessen, 2014: 79). The term Subject generally refers to the grammatical Subject, a construct rooted in the Indo-European grammatical tradition that integrates semantic, pragmatic and morpho-syntactic dimensions (Song, 2018: 9). In English, the Subject and Predicate are the two basic components of a simple clause. The Subject is the component that defines the topic of the sentence, i.e., what the clause is about. It is typically a nominal group, precedes the verb, and may assume the ergative case, among other characteristics (Quirk et al., 1985: 724–726). This illustrates that the Subject is not a self-contained grammatical concept that encapsulates both form and meaning. Given the linguistic disparities between Chinese and Indo-European languages, a majority of scholars in Chinese linguistics propose that the concept of Subject should be recalibrated for the analysis of Mandarin. They emphasize the principle of “integration of both form and meaning” in defining the subject (Lü, 2002: 514), that is, taking both the semantic and pragmatic standpoints and word order into consideration. However, a consensus for applying this principle to the process of Subject identification has yet to be established. The crux of the construction under discussion can be ascribed to this, which underscores the tension between semantics and pragmatics, as well as between semantic content and word order.

CG holds that the Subject pertains to the formal level, specifically the syntactic category, and stands as one of the pivotal components of clause structure, often conflated with a PR (Fawcett, 1999, 2008: 51). Since a PR is usually expected by the Process, any clause component that is expected by the Process, irrespective of its formal realization, is either a Subject or a Complement. In transitivity, the semantic structure of a clause is generally “Participant + Process + Participant,” with the corresponding syntactic structure being “S ^ M ^ C.” In CG, it is contended that a PR is typically realized by a nominal group or a quality group (adjectives and adverbs), while a CR is typically realized by a prepositional group. Fawcett (2008: 142, 143) notes that a PR is not necessarily a “thing” or “quality,” rather it may be “place,” “direction,” “location,” “time,” etc. So, a PR can also be used to answer the question “where,” and “when.” This is because PRs are all inherent in the Process. The removal of a PR results in an incomplete clause meaning. For instance, in the clause of *Ivy lives in Cardiff*, the underlined segment is a Complement, not an Adjunct, as it represents a PR expected by the Process of “live.” Although *in Cardiff* takes the form of a prepositional group, it maintains its semantic role as a Participant. As a formal category in CG, the term Subject has a variety of functions rather than being confined to a single form that realizes interpersonal meaning, as described by Halliday and Matthiessen (2014: 139). Specifically, the Subject, in conjunction with the Main Verb and the Complement, realizes the experiential meaning of the clause and shapes the transitivity structure. The Subject, together with the Operator (Finite), realizes interpersonal meaning of the clause and forms the mood structure. Furthermore, as the Subject is typically positioned at the beginning of the clause and states what the clause is about, any Subject that conflates with a PR functions as the Subject Theme.

In the framework of CG, the identification of the English Subject is guided by the Mood Test (Fawcett, 2008: 66), which is conducted in three steps. The first step is to find the Operator. The Operator is either a modal verb such as *may*, *shall*, *can* or one of a small number of other verbs. The second step is to re-express the “information giver” (declarative) as an “information seeker” (interrogative). The last step is to identify the Subject, that is, the Subject is the item which, by occurring before or after the Operator, tells you whether the clause is an “information giver” or an “information seeker.” The Concept of the Subject in CG offers at least four implications for understanding the Subject in Mandarin: (i) the Subject is intricately linked to the semantics of the clause verb, suggesting that context should be a factor in Subject identification due to the variable meanings verbs may assume in different contexts; (ii) the Subject is typically conflated with a PR, indicating that PRs within the transitivity structure should be taken into account during the identification process; (iii) the Subject is typically the Subject Theme, positioned at the beginning of the clause, implying that word order of the structural components of the clause must be considered; (iv) there is only one Subject in each clause capable of realizing multiple functions, negating the possibility of multiple Subjects.

Adhering to the methodological principle of “integration of both form and meaning” in grammatical analysis maintained in Chinese linguistic tradition, we propose that the recognition of the Subject in Mandarin should integrate both semantic functions and word order (position). Drawing from CG, the general rule for Subject identification in Mandarin is that if a word is a leading Participant expected by the Process and prior to the Predicator, it is deemed the Subject. Conversely, if a word does not fulfill the role of a leading Participant, it is a Complement, even if it precedes the Predicator. By “leading Participant,” we mean logically and broadly the “doer of the action” in the Performer’s decision making, encompassing the Agent in Action Processes, the Carrier in Relational Processes, the Sensor in Mental Processes, etc. One of the difficulties for the Subject identification in Mandarin is the case where two PRs precede the verb. For instance, in the clause of *Jiu wo bu he* (“Wine, I do not drink”), *jiu* (“wine”) and *wo* (“I”) are two PRs preceding the verb. In this case, we have to resort to the belief system with the underlying transitivity structure, i.e., the clause logically expresses the meaning “Wo he jiu” (“I drink wine”). In other words, *wo* (“I”) is the leading PR. Therefore, in this clause *wo* (“I”) is the Subject rather than *jiu* (“wine”), because *wo* is the leading participant in the process of “he” (“drink”) and is situated before the Predicator *he* (“drink”). The leading participant is strictly contingent upon the meaning of the verb. If the same verb (i.e., in the same form) expresses different Processes, the leading Participant may vary accordingly. In other words, the principle of “integration of both form and meaning” should be maintained in Subject identification. According to Fawcett (2013: 125), the decision-making process involves a higher component (i.e., belief system) which causes the appropriate choices to be made in the system networks for TRANSITIVITY, MOOD, etc. Therefore, theoretically any PRs may have the potential to become the leading Participant in specific linguistic constructions within the Performer’s decision making process.

### 5.2 The functional syntactic analysis of the construction

**Figure 3:**
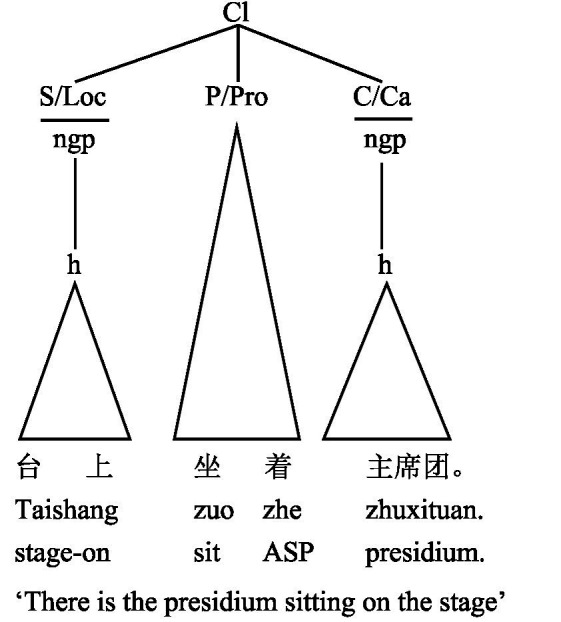
The functional syntax of the construction of “Taishang zuo-zhe zhuxituan”.

The cardinal principle in CG is that meaning potential is the generative basis for linguistic forms (Fawcett, 2008: 40). Therefore, the syntactic description in CG is meaning-centered. Given that *taishang* in the construction is a PR as analyzed above, the Adjunct analysis must be dismissed. The question then is whether it should be analyzed as a Subject or a Complement. In light of aforementioned criteria for the Subject identification, since *taishang* precedes the Predicator *zuo-zhe* and the construction logically conveys the meaning of “somewhere sits someone,” *taishang* is the leading PR. Therefore, it is *taishang* that qualifies as the Subject in this construction. This analysis is echoed by He (2016) in her analysis of the Subject in Chinese existential constructions based on CG. Based on the above understanding, the functional syntax of the construction of “Taishang zuo-zhe zhuxituan” can be illustrated as Figure 3.

As we can see from Figure 3, the construction syntactically has three key elements: a Subject, a Predicator, and a Complement. The Subject is conflated with the PR of Location and filled by the nominal group *taishang*, which has only one element, i.e., the head. The predicator is conflated with the process, i.e., locational relational process. The complement is conflated with the PR of carrier, which is filled by the nominal group *zhuxituan*, and it has only one element, i.e., the head. The item expounded by the aspect particle *zhe* is part of the predicator.

As for the standards of grammatical analysis in Mandarin, Lü (2002: 542) emphasizes that they should be simple, specific, easy to base, and a little flexible. The merit of the functional syntactic analysis presented herein lies in its adherence to the principle of “integration of both form and meaning,” a tenet that is deeply ingrained in the Chinese linguistic tradition. This analysis is underpinned by two observations: one is that the locative *taishang* is the leading PR in the transitivity structure, and the other is that Mandarin is characterized as having the basic syntactic order of “S ^ P ^ C” among verbal clauses (Chao, 1968: 94; Li and Thompson, 1981: 23). Therefore, from the perspective of CG there is no semantic and syntactic mismatch in this construction. The essence of this so-called “locative inversion” construction resides in the re-working or re-organization of the relationship between semantic choice and the lexicogrammatical potential for the realization of semantic choice. To concretely elucidate the strengths of our syntactic analysis, we will briefly compare it with alternative approaches stated in Section 2.

In contrast to the Adjunct view, our analysis admits the PR status of *taishang*, thereby affirming that this construction constitutes a unique syntactic pattern of its own. In CG’s term, it forms its own PR configuration and is realized by a different syntax. The consequence is that it challenges the conventional idea that syntactic element of Adjuncts are to locatives or prepositional groups. In fact, Quirk et al. (1985: 60/658) also explicitly note that Subjects and Complements can be realized by prepositional groups other than nominal groups. For instance, the underlined prepositional groups in (7) all serve as Subjects. In the English language, the Subject may occasionally follow the Main Verb in specific contexts, exemplified by the so-called full inversion (see the underlined part in (8)). In other words, the Subject retains its function as the Subject and is not subsumed by the Complement, primarily due to the agreement rule required for the Subject and the Main Verb. However, in Mandarin there is no such agreement rule in the Subject description, i.e., lacking of inflectional variations, and word order thus plays the pivotal factor in distinguishing one syntactic element from another. Even the same semantics may resort to different linguistic forms. From the perspective of multi-functional approach endorsed by SFL, especially CG, while certain constructions might share similar experiential structures, their informational and thematic meanings can diverge. This implies that different constructions fulfill distinct functions across various contexts to meet disparate communicative demands. Therefore, it is not particularly compelling to regard the construction of “Taishang zuo-zhe zhuxituan” as merely an inversion of (5) or as a variant of (6), as is done in other linguistic approaches.

(7) a. On Tuesday will be fine. (Quirk et al., 1985: 658)

b. During the vacation is what we decided. (Quirk et al., 1985: 658)

(8) a. In the doorway stood my brother. (Quirk et al., 1985: 522)

b. On the very top of the hill lives a hermit. (Quirk et al., 1985: 522)

Compared with the Subject view, while there appears to be a superficial alignment with our syntactic analysis—specifically, the shared view that *taishang* functions as the Subject—there are actually essential differences in the identification of the Subject. The Subject view bases on the linguistic form alone, i.e., word order, to determine the Subject status of *taishang*. This viewpoint could inadvertently propagate the misconception that all nouns preceding the verbs automatically qualify as Subjects. Our analysis, however, is predicated on a meaning-centered approach to identifying the Subject, complemented by an appropriate consideration of linguistic form—*taishang* is recognized as a PR that appears before the verb. Within the framework of CG, the underlined part in (9), despite their varied linguistic forms, are all considered Subjects, as they all represent inherent PRs that are expected by the process of *you* (“have”) and are positioned before the verb *you*. This analysis aligns with the arguments presented by He (2016) and Song (1991: 124).

(9) a. Beiing you zuo gugong (“Beijing has a Palace Museum”).

b. Beijing chengli you zuo gugong (“There is a Palace Museum in Beijing city”).

c. Zai Beijing chengli you zuo gugong (“In Beijing city, there is a Palace Museum”).

The Big Subject view encapsulates the ongoing debate between “Subject-Predicate” and “Topic-Comment” frameworks in Mandarin description. Some scholars, with a more radical stance, contend that Mandarin should be classified as a “Topic-Comment” language, advocating for the abandonment of the “Subject-Predicate” model due to the perceived negligible role of the Subject in Mandarin syntax (Li and Thompson, 1981: 19; Shen, 2017). However, from CG’s standpoints, this view seems to overlook the multi-stratal nature of language, necessitating distinct descriptions at different levels. Consequently, this view confuses semantic and syntactic structures. Zhu (2001: 409) has strongly criticized this practice. As highlighted by Peng (2018: 214), the concept of the subject, while originating from the Indo-European grammatical tradition, retains certain universal categorizations that are applicable across languages. It is our conviction that it remains essential to uphold the concept of the Subject in Mandarin. Therefore, from the perspective of CG, treating *taishang* as the Big Subject disrupts the semantic and syntactic integrity of the construction in question. The ramification of such an approach may lead to a significant expansion of what is termed “subject-predicate predicate clauses” in Mandarin.

The Complement view recognizes that *taishang* is a PR, thereby recognizing the semantic relationship between the Predicator *zuo-zhe* and the locative *taishang*. However, this view persists in denying *taishang* the status of the Subject, instead considering only *zhuxituan* as the Subject. This stance may stem from the misguided assumption that *zhuxituan* still retains the role of Agent, given that Agents are conventionally seen as the default Subjects across world languages in traditional frameworks. By designating *taishang* as the Subject and *zhuxituan* as the Complement, our analysis might conflict with the traditional analyses that prioritize Agenthood as the primary criterion for Subjecthood, and challenges the conventional expectation that Subjects are typically nominal entities performing actions. However, the benefits of this approach within CG are significant, as CG emphasizes that the Subject is determined by both form (word order) and meaning (PRs). By recognizing *taishang* as the Subject, CG aligns with the principle of “integration of both form and meaning” maintained in Mandarin linguistic tradition. The locative *taishang* occupies the initial position, a key indicator of Subjecthood in Mandarin, and serves as the Theme of the clause, setting the scene for the discourse. This analysis captures the functional motivation behind the construction, which is to highlight the location as the starting point of the message. Treating *zhuxituan* as a complement (carrier) reflects its role as the entity whose existence is being stated, adhering to the relational process framework in CG where locations and carriers are key PRs. Thus, from the perspective of CG, the complement view seems to ignore the significance of word order as a crucial linguistic form in realizing meanings in Mandarin, which is deemed partially functional.

All in all, the functional syntactic analysis of this construction within the theoretical framework of CG presents a functionally motivated perspective that prioritizes discourse needs and semantic roles over traditional agent-subject alignment. While it risks contradicting conventional syntactic analyses, its strength lies in offering a more integrate form-meaning account tailored to Mandarin’s linguistic structure. Ultimately, our analysis enriches Mandarin syntax by bridging Hallidayan functionalism with Mandarin’s topic-comment pragmatics, offering a template for analyzing other “special” constructions in discourse-oriented languages.

## 6 Functional motivations of the construction

Within SFL, every aspect of grammar is intrinsically linked to the communicative role of language. A text-sentence should not be examined in isolation from the discourse that frames it, given that the choices made by a Performer in the context of the discourse momentarily become part of the short-term knowledge base that influences the semantic decisions within the text-sentence (Fawcett, 1980: 90). An in-depth investigation of the BCC corpus data, together with a review of pertinent scholarly works in the literature, reveals that there are a minimum of three functional motivations that underpin the usage of this particular construction.

The first functional motivation is to make *taishang* the subject theme. Thematic meanings are linguistic meanings between which a user of a language chooses in order to serve the various purposes that may arise in a developing discourse (Huang, 2017: 164). In CG, different types of theme are recognized, including subject theme, enhanced theme, marked PR theme, etc. The system of Subject Theme is dependent on the system of TRANSITIVITY (Fawcett, 2011), since the grammar is not in a position to state which elements are available to be chosen for presentation as the subject theme until it knows which PRs have been selected and which PRs will actually be present in the clause. Fawcett (2008: 109) claims that the subject theme of a clause is the aspect of the meaning of a typical subject that tells the addressee “what the clause is about.” In the construction “Taishang zuo-zhe zhuxituan,” *taishang*, as the PR of location, signifies that the focus of the clause is on the location, specifically *taishang*, rather than some other places. It is natural then for *taishang* rather than *zhuxituan* to be designated as the subject theme. This construction essentially serves as a description of *taishang*, utilizing the entire clause to highlight the event occurring on the stage. Consequently, this construction is more appropriately employed to address inquiries such as *What about taishang?* This distinction further elucidates the divergence between this construction and (5), where *zhuxituan* assumes the subject theme. In other words, (5) is about *zhuxituan* rather than *taishang*. It is more appropriate to answer the question of *What about zhuxituan?*

The second functional motivation is to set the scene for the discourse, i.e., representing background information. If we analyze the semantics and syntax of a clause just from the perspective of the clause itself, the result may not be so satisfactory, because the natural environment of a clause is not standing alone as a numbered example, but as an element in the discourse structure of a longer text (Fawcett, 2008: 39). As argued by Song (1991: 98), this construction typically serves descriptive rather than narrative function, a usage frequently encountered in the descriptive segments of operas or environmental depictions in novels. Through corpus investigation, it reveals that this construction is particularly appropriate for scene-setting, since the employment of locatives as the point of departure is a conventional method for environmental description. Consider the underlined clause in (10) from the BCC, which illustrates this point effectively.

(10) *Chuangwai fang-zhe yanhuo, meiyixiang dou rang wodexin yinyinzuotong* (“There are fireworks setting off outside the window, and every sound hurt my heart”). (BCC)

In (10), the underlined clause provides the background information, offering a rationale that sets the scene for the subsequent clause. Of the 416 samples we collected in the BCC featuring the pattern of “location + zuo-zhe + thing,” a substantial 76% are utilized for scene-setting purposes. From a functional perspective, it can be argued that particular syntactic patterns often arise from verbs that appear with high frequency. In other words, there is a tendency that the more frequently-used verbs are employed, the greater the likelihood of their transition from foreground to background usage, potentially culminating in innovative applications. In this construction, it is precisely because of the frequent use of *zuo-zhe* in Mandarin that the so-called “locative inversion” usage of this construction is generated. Upon the investigation of the corpus data in the BCC, it reveals that verbs within the “location + zuo-zhe + thing” pattern exhibit similar functional characteristics. These clauses are usually used as “scene-setting” clauses in attributive or conditional constructions by providing background information for the whole discourse. It is therefore an important discourse purpose for the construction to set the scene in longer texts.

The third functional motivation is to enhance the state of “existence” associated with *zhuxituan*. As previously discussed, numerous scholars categorize this construction within the realm of existential clauses. From the perspective of CG, existential clauses fall under the umbrella of Relational Processes, because locational and existential clauses share PRs. Given this categorization, one might question why the Performer does not simply employ the conventional existential clause introduced by the verb *you* (“have”), as in *Taishang you zhuxituan* (“There exists the presidium on the stage”). Song (1991: 100) argues that the quintessential existential clauses, led by *you* in Mandarin, are utilized expressly and merely to denote the existence of entities, rather than the state of their existence. Therefore, this construction, by means of amalgamating the verb with the aspect particle *zhe*, forms a distinctive pattern designed to portray the state of existence of certain entities, be they static or dynamic. In other words, *zuo-zhe* is employed to vividly describe the static state in which the entity *zhuxituan* exists, as opposed to *zhan-zhe* (“stand”) or something like that. To accentuate the existential state of *zhuxituan*, native Mandarin speakers tend to use this construction when the existence of *zhuxituan* is of particular interest. Functionally, it is the overall syntactic pattern that determines the meaning of the construction. Thus, different verbs in the “V-zhe” formation are harnessed to accentuate different processes. Song (1991: 99) further argues that verbs within the “location + zuo-zhe + thing” pattern can be bifurcated into static and dynamic. The two categories of verbs are used to describe distinctive states. Let us compare the construction of “Taishang zuo-zhe zhuxituan” with (11) to elucidate these points.

**Table 6:** 

(11)	台上	唱	着	黄梅	戏。
	Taishang	chang	zhe	huangmei	xi.
	stage-on	sing	ASP	Huangmei	opera.
	“There is the Huangmei opera singing on the stage”.				

In (11), the verb *chang* (“sing”) plus the Aspect particle *zhe* serves to emphasize the dynamic state of existence of the Huangmei opera, signifying that it is currently being performed on the stage. In contrast, “Taishang zuo-zhe zhuxituan” emphasizes the static state of existence of the presidium. This distinction illustrates that in evaluating the meanings of structures, we need to take account of the words that expound the elements as well as the syntax of the elements, as divergent verbal meanings can engender distinct communicative functions. The construction in question is used to convey the performer’s intended meaning—specifically, the state of existence—in a manner that is more straightforward, expedient, and natural. It further proves that this construction is not an inversion of (5) or (6), and that word order is an important grammatical-rhetoric device in Mandarin, as Chen (1984: 56) has compellingly argued.

## 7 Conclusion

In this study, we investigated the construction of “Taishang zuo-zhe zhuxituan” in Mandarin from a CG approach. The emerging dispute with regard to the grammatical function of the locative *taishang* in this construction can be attributed to the fact that few studies have investigated the construction from a form-meaning matching perspective. In light of this, we argue that a comprehensive study of a linguistic construction should take both semantics and syntax into consideration, and a holistic investigation must also account for functional motivations, aligning with the Performer’s discourse purposes.

Adhering to CG’s functional linguistic framework, we then examined the Process and PRs, functional syntax, and functional motivations of the construction. The findings show that the construction expresses a locational relational process, with *taishang* expressing the PR of location, *zuo-zhe* expressing the process, and *zhuxituan* expressing the PR of carrier. The PR configuration of this construction distinguishes itself from those of other similar constructions, such as “Zhuxituan zuo zaitaishang” (“The presidium sits on the stage”) and “Zhuxituan zaitaishang zuo-zhe” (“On the stage, the presidium sits”). The functional syntax of this construction is “S ^ P ^ C”, where the subject is conflated with the PR of location, the predicator with the process, the complement with the carrier, and *zhe* after the verb *zuo* is an aspect particle which is part of the predicator. The functional motivations underpinning the usage of this construction are multifaceted: to make *taishang* the subject theme, to set the scene for the discourse, and to enhance the state of existence of *zhuxituan*. The study further substantiates that there is no semantic and syntactic mismatch within the construction, dispelling the notion that it represents a “locative inversion” construction.

This study’s analysis of the Mandarin existential construction “Taishang zuo-zhe zhuxituan” within the CG framework elucidates its syntactic-semantic dynamics, resolving longstanding debates by redefining the locative *taishang* as a subject theme and underscoring the role of the aspect marker *zhe* in encoding durative states rather than mere existence. The CG approach demonstrates Mandarin’s syntactic flexibility, where locatives assume Subject roles to prioritize thematic prominence and scene-setting, reflecting the language’s topic-comment orientation (Li and Thompson, 1981). By reclassifying *zhuxituan* as a Complement conflated with the carrier role—rather than a “delayed Subject”—this analysis challenges traditional formal syntax paradigms, offering a unified framework for similar constructions (e.g., *Qiangshang gua-zhe yifu hua*). The findings reveal existentiality in Mandarin as inherently process-oriented, blending relational and material processes through verbs like *zuo* (“sit”) or *gua* (“hang”), which fuse existence with dynamic or static states. These insights hold significant pedagogical value for L2 instruction: contrastive analysis with English existential structures (e.g., *there is/are*) can address common learner errors, such as omitting *zhe* or misplacing locatives, while targeted exercises on aspectual awareness and scene-setting narratives can enhance communicative competence. Beyond pedagogy, the study identifies critical future research avenues, including cross-construction analysis to explain verb frequency hierarchies (e.g., why *zhan* (“stand”) is common but *pao* (“run”) is rare), dialectal comparisons (e.g., Cantonese’s *jau^5^* vs. Mandarin’s *zhe*), typologically distinct languages (e.g., Japanese-te *iru* constructions) to identify universal and language-specific features, and psycholinguistic experiments to validate native speakers’ cognitive processing of thematic roles. Computational applications, such as integrating CG principles into natural language processing tools, could improve parsing accuracy for non-canonical structures. Theoretically, CG’s semantically grounded syntax bridges SFL and cognitive approaches, illustrating how functional motivations shape syntactic choices—a perspective pivotal for rethinking Mandarin beyond Eurocentric models. By advocating corpus-driven, multifunctional methodologies, this study underscores the richness of Chinese existential constructions and the need to embrace multiplicity in grammatical analysis. While the CG-based interpretation remains exploratory, it opens pathways for redefining syntactic ambiguity, emphasizing the interplay of form, meaning, and context in Mandarin and beyond. Ultimately, this work contributes to a broader understanding of how languages grammaticize existence, offering a template for cross-linguistic studies and reinforcing CG’s utility in addressing the complexity of “special” constructions in typologically diverse languages.

## Data Availability

The original contributions presented in the study are included in the article/supplementary material, further inquiries can be directed to the corresponding author.

## Footnotes

^1^Keys used in this study: V = verb; ASP = Aspect particle; Cl = Clause; S=Subject; M = Main Verb; C=Complement; O=Operator; P=Predicator; X = Auxiliary; A = Adjunct; PR = Participant Role; CR = Circumstantial Role; Pro = Process; Ag = Agent; Ca = Carrier; Loc = Location; Ag-Ca = Agent-Carrier; Af-Ca = Affected Carrier; ngp = nominal group; h = head; dd = deictic determiner; ^=followed by; /=conflated with; | = consist of; — = filled by; △ = expounded by. Note that following the systemic functional tradition, names of structural functions are spelt with an initial capital and names of systems with upper case.

^2^In Mandarin Chinese, the item *zhe* (着) has various functions in clauses, such as expressing the continuous aspect, representing the continuation of state, emphasizing the imperative mood, etc. For the sake of simplicity, this paper just glosses it as an Aspect particle (see Song, 1991; He, 2016; Li, 2011).

^3^Note that we use the term “Predicator,” not “Predicate.” The latter term has been used in traditional grammar, formal grammar (where it is roughly equivalent to VP, or Verb Phrase). It should be noted that CG uses the term Main Verb. Considering that the Process meaning in Mandarin Chinese can be realized not only by verbs but also by nouns, adjectives, and other items, we, following Halliday and Matthiessen (2014: 151), use the term “Predicator.”

^4^Some scholars differentiate “Object” from “Complement” especially in the traditional grammar, but in SFL the division is not needed, since the distinction between them can be modelled in the explicitly semantic categories of the types of PRs. Following the systemic tradition, we will unify the two terms as Complement for convenience.

^5^Some may claim that we cannot simply use the test for English to prove a Chinese structure. Our claim is that the test at least is comparable to Chinese experientially, since transitivity is universal almost in all world languages.
